# Educating Health Professionals about Drug and Device Promotion: Authors' Reply

**DOI:** 10.1371/journal.pmed.0040088

**Published:** 2007-02-27

**Authors:** Peter R Mansfield, Jerome R Hoffman, Joel Lexchin

We thank Prof. Ravi Shankar for his thoughtful reflections [1] on our recommendations for education about drug and device promotion [2]. Prof. Shankar is one of the few who have published an evaluation of education about drug promotion [3], so we are pleased that he concurs with most of our recommendations. We agree with him that implementation of our recommendations will be difficult. The main barrier to implementation is the widespread denial by health professionals that we are often adversely influenced by promotion. This denial arises partly from ignorance of the evidence about promotion and partly from a refusal to accept that evidence, because it is viewed as insulting our self-esteem [4]. We believe health professionals need to move from overconfident illusions of invulnerability to accept that we are human, so it is normal for us to be misled by persuasive promotional techniques [5].

Prof. Shankar seems to believe that doctors can be taught to “critically evaluate drug promotion” [1] so as to “optimise time spent with medical representatives” [3], although he acknowledges only “mixed success” with his medical students [1]. By contrast, we are pessimistic that doctors will ever be able to gain more good than harm from visits by sales representatives in any country. All the relevant evidence of which we are aware suggests that exposure to promotion correlates with less appropriate prescribing [6]. Furthermore, the skills required to avoid being misled may take many years to learn, and many hours to apply after each visit. There is no proven method for overcoming normal human vulnerabilities such as the tendency to believe attractive, socially skilled people, especially if they have built up trust over many visits. Consequently, we believe the onus is on anyone who claims that it is possible for health professionals to learn how to gain net benefit from sales representatives' visits to produce evidence to support that claim.

Prof. Shankar is ambivalent about our recommendation that health professionals avoid promotion because in his country, Nepal, sales representatives “may be their only source of information about medicines.” We share Shankar's concern about lack of relevant independent information for doctors in many countries. In all countries, whoever pays for health care will get better results at lower cost by funding independent information, rather than paying direct and indirect costs from inappropriate drug and device use caused by misleading promotion. Payers for health care are more likely to fund such initiatives if they understand how harmful drug promotion is. Even if no such initiatives are forthcoming, we reject the idea that any information—even if it is misleading—is better than no information at all. The majority of new drugs are more expensive than their older analogues, but no better (and sometimes worse). Patients would be better off if doctors were ignorant non-prescribers of such drugs rather than misinformed frequent prescribers.

Current patterns of health professionals' interactions with the drug and device industry are causing much harm [6]. Our three main tasks are to develop optimal recommendations for improvement, identify barriers to implementation, and develop methods for overcoming those barriers. We appreciate any input, including Prof. Shankar's reflections, that will help us improve our performance on those tasks.
